# Mixed type of total anomalous pulmonary venous connection: diagnosis, surgical approach and outcomes

**DOI:** 10.1186/s13019-020-01332-7

**Published:** 2020-10-02

**Authors:** Ming Xiang, Chun Wu, Zhengxia Pan, Quan Wang, Linyun Xi

**Affiliations:** 1 Department of Cardiothoracic Surgery, Children’s Hospital of Chongqing Medical University, Chongqing, PR China; 2 Ministry of Education Key Laboratory of Child Development and Disorders, Children’s Hospital of Chongqing Medical University, Chongqing, PR China; 3 National Clinical Research Center for Child Health and Disorders(Chongqing), Children’s Hospital of Chongqing Medical University, Chongqing, PR China; 4 China International Science and Technology Cooperation base of Child development and Critical Disorders, Children’s Hospital of Chongqing Medical University, Chongqing, PR China; 5 Chongqing Key Laboratory of Pediatrics, No.136, Zhongshan 2nd Road, Yuzhong Dis, Chongqing, 400014 China

**Keywords:** Pulmonary vein, Mixed type, Mortality

## Abstract

**Purpose:**

To summarize the diagnosis and treatment of 13 patients with mixed-type total anomalous pulmonary venous connection (TAPVC) and propose another classification for mixed TAPVC.

**Methods:**

A retrospective review of 13 patients with mixed TAPVC undergoing repair at a single institution was conducted between January 2010 and November 2019. The diagnosis of mixed-type TAPVC was made in all patients using echocardiography combined with computed tomography angiography. According to the mixed TAPVC anatomy, there were 3 patients with type I TAPVC (2 + 2 veins), 10 patients with type II TAPVC (3 + 1 veins) and no patients with type III TAPVC. Correspondingly, there was 1 patient with the “SVC + VV” subtype, 2 patients with the “CS + C” subtype, 8 patients with the “CS + VV” subtype, 1 patient with the “CS + SVC” subtype and 1 patient with the “RA + SVC” subtype according to our classification system. All patients underwent cardiopulmonary bypass surgery.

**Results:**

The median weight at surgery was 4.6 ± 1.0 kg (3.4–7.3 kg), and the median age at surgery was 96.2 ± 81.2 days (10–242 days). The median cardiopulmonary bypass time was 132.7 ± 25.1 min (range, 100 to 190 min). The cross-clamping time was 69.2 ± 14.4 min (range, 45 to 88 min). The hospital mortality rate was 7.7% (1 of 13), with late mortality occurring in 1 patient because of pulmonary venous obstruction (PVO) 7 months after surgery. The average follow-up after surgery was 3.4 ± 2.2 years (range, 5 months to 8 years). The survival rates at 3 and 5 years were both 90.9% ± 8.7% (95% CI: 73.8–108%). All remaining surviving patients were asymptomatic.

**Conclusion:**

Mixed TAPVC can be repaired with good results in children and can be correctly diagnosed with echocardiography combined with computed tomography angiography. The classification system we propose is pragmatic and can guide the surgical approach.

## Introduction

Total anomalous pulmonary venous connection (TAPVC) is divided into supracardiac, cardiac, infracardiac and mixed types [1]. With the accumulation of experience in surgical treatment and intensive postoperative care, the outcomes of TAPVC have improved over time, with reported mortality rates consistently < 10% [2]. However, mixed-type TAPVC, in which more than one level of pulmonary venous drainage exists, seems to be the most problematic subgroup. In some series, the mortality rate of patients with mixed TAPVC is still high [2], but because of the rarity of this condition, few reports have focused exclusively on this subtype, and it is difficult to derive enough experience regarding surgery. A useful classification system for mixed type TAPVC that can indicate a suitable surgical approach is difficult to formulate due to the variability of the pulmonary venous connections, although one classification for subtypes of mixed TAPVC has been proposed [3, 4]. The diagnosis and treatment of mixed TAPVC are still challenging and can be daunting. The aim of the present study was to retrospectively assess the results and to share our experience in diagnosing and surgically treating several cases of mixed TAPVC and propose another classification that can provide information related to the selection of the surgical method.

## Patients and methods

TAPVC can be classified into 4 subtypes, and the mixed type is the rarest form, accounting for only 5–10% of TAPVC cases [3]. The surgical approach varies for different types of mixed TAPVC. Accurate diagnosis, especially regarding how all four pulmonary veins drain, has been emphasized as a critical factor of achieving improved results. We referenced the classification of mixed TAPVC subtypes proposed by Chowdhury et al. [4] and propose another classification based on our experience.

**Fig. 1:**
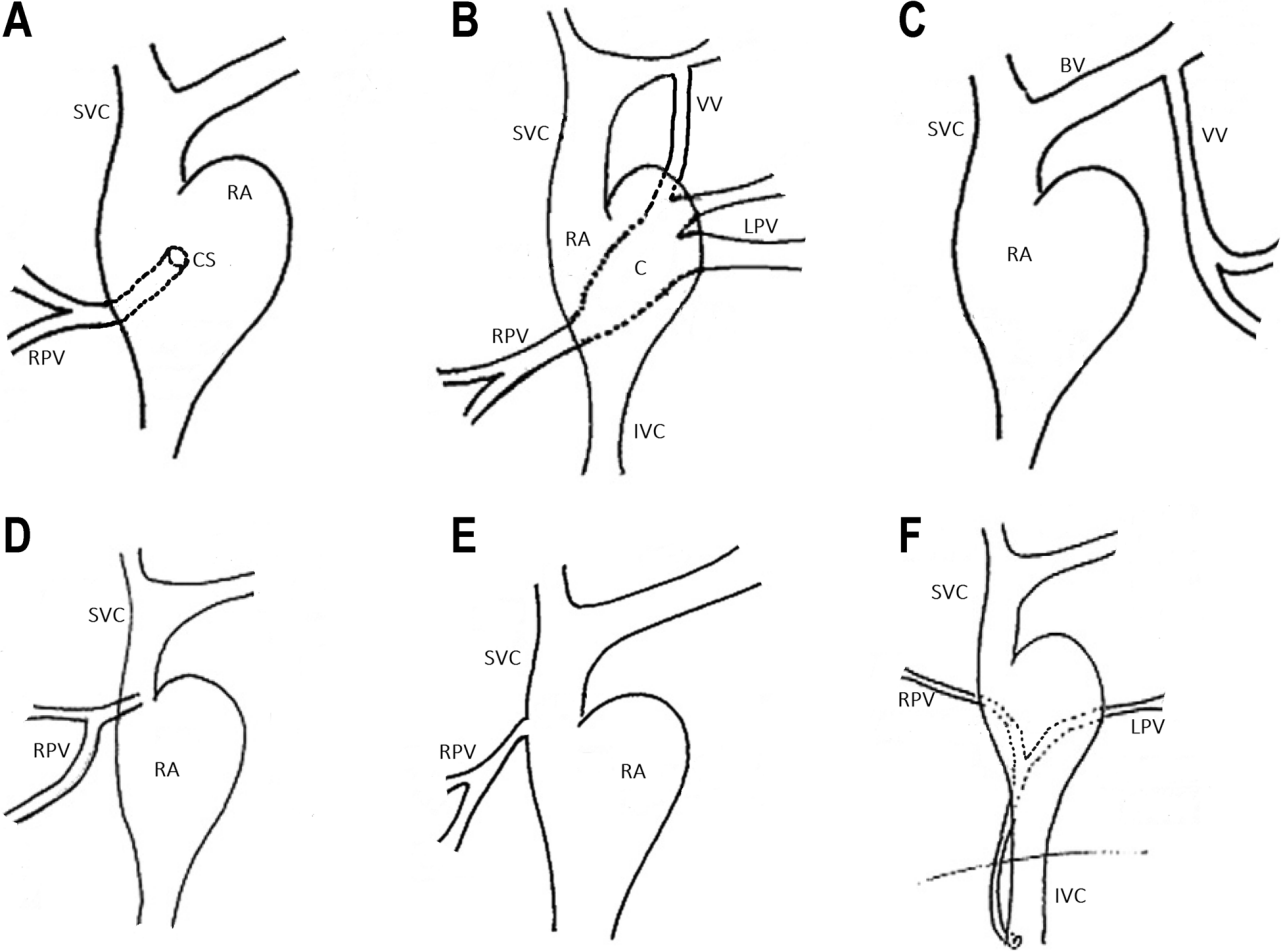
**a** to **f** are the illustrations of the first to the sixth classification system.the pulmonary vein drainage in the illustrations (RPV or LPV) is variable, the pulmonary venous drainage sites such as CS,RA and SVC are the key points. CS: Coronary sinus;C: Confluence;VV:vertical vein;IVC: inferior vena cava;SVC: the superior vena cava;RA: Right atrium; LPV, left pulmonary vein; RPV, right pulmonary vein; BV, brachiocephalic vein

The classification we propose is based on each site of pulmonary vein drainage into the systemic venous system and how all four pulmonary veins drain, such as whether a site of pulmonary venous confluence is present, which veins drain to the confluent site. We analyzed the published literature [1–4] focusing on the morphology of the mixed subtypes of TAPVC and summarized the sites of the pulmonary veins. The pulmonary venous drainage sites were always as follows: (1) Coronary sinus (CS): In this group, one or more pulmonary veins are connected to the CS, and the key point of this group is the site of the drainage rather than whether the pulmonary veins form a confluent site or the number of pulmonary veins and which pulmonary vein drainage to the coronary sinus. (2) Confluence (C): In this group, two or more pulmonary veins form a confluent site, which is then connected to the vertical vein (VV), the superior vena cava (SVC) or inferior vena cava (IVC). The key point of this group is that a confluent site or common chamber is formed. (3) VV: Usually one pulmonary vein drains to the brachiocephalic vein via the VV. The key point is that no confluent site or common chamber is formed and the blood drains to the brachiocephalic vein. (4) Right atrium (RA): Pulmonary veins are connected directly to the RA. (5) SVC: One or more pulmonary vein drains to the SVC, not the brachiocephalic vein, without forming a confluent site or common chamber. (6) IVC: One or more pulmonary vein drains to the IVC without forming a confluent site. (7) Bizarre anatomical variants (BAVs): In this group, the anatomical variants were bizarre and required pulmonary venous rechanneling with individualized surgical techniques. The classification we propose is based on the various combinations of drainage sites, such as “CS + C” or “SVC + RA + CS” [5](Fig. 1).

In this retrospective analysis, we reviewed the clinical records of patients who underwent repair for mixed TAPVC between January 2010 and November 2019. All of these patients underwent biventricular correction. Patients with a single ventricle, atrial isomerism and other complex congenital heart diseases were excluded. Patient characteristics, such as weight and age comorbidities, pulmonary venous anatomy, demographics, and clinical, operative, postoperative, and follow-up data were recorded.

A total of 231 patients who were diagnosed with TAPVC underwent surgical repair at the Children’s Hospital of Chongqing Medical University. Thirteen patients (5.6%) had mixed TAPVC; of them, 11 were male, and 2 were female. The median weight at surgery was 4.6 ± 1.0 kg (3.4–7.3 kg), and the median age at surgery was 96.2 ± 81.2 days (10–242 days). Ten patients were younger than 6 months, including 3 neonates. Preoperative PVO occurred in 3 patients (Table 1).

**Table 1:** Characteristics and distribution of mixed TAPVC patients

Type	N (%)	Sex (M/F)	Age at surgery (days)	Weight at surgery (kg)	Preoperative PVO
Mixed	13 (5.6%)	11/2	96.2 ± 81.2	4.6 ± 1.0	3 (23.1%)

The pattern distribution among the 13 patients is shown in Table 2. Three patients (23.1%) had type I TAPVC. In this subtype, the right pulmonary veins in 2 patients (the fourth and twelfth patients) connected to the CS and left superior and inferior veins to form a site of confluence with the brachiocephalic vein via a VV (“CS + C” subtype in our classification system); in the other patient (the eighth patient), the right superior and inferior veins formed a site of confluence and connected to the IVC, while the left superior and inferior veins formed a site of confluence and connected to the brachiocephalic vein via a VV (“C + C” subtype in our classification system). 10 (76.9%) patients had type II TAPVC. In this group, the right pulmonary vein and the left inferior pulmonary vein in 8 (80%) patients connected to the CS, and the left superior pulmonary vein connected to the brachiocephalic vein via a VV (“CS + VV” subtype in our classification system). In 2 patients, the left pulmonary vein and the right inferior pulmonary vein connected to the RA, and the right superior pulmonary vein connected to the SVC (“RA + SVC” subtype in our classification system).

**Table 2:** Pulmonary venous drainage patterns in mixed TAPVC

Patients	Mixed type	Subtypes	Sites of drainage (right)	Sites of drainage (left)
1	CS + VV	Type II	RIPV, RSPV and LIPV to SC	LSPV to brachiocephalic vein via VV
2	SVC + RA	Type II	RSPV to SVC	LIPV, LSPV and RIPV to RA
3	CS + VV	Type II	RIPV, RSPV and LIPV to SC	LSPV to brachiocephalic vein via VV
4	CS + C	Type I	RIPV and RSPV to CS	LIPV and LSPV to a confluence to brachiocephalic vein via VV
5	CS + VV	Type II	RIPV, RSPV and LIPV to SC	LSPV to brachiocephalic vein via VV
6	CS + VV	Type II	RIPV, RSPV and LIPV to SC	LSPV to brachiocephalic vein via VV
7	CS + VV	Type II	RIPV, RSPV and LIPV to SC	LSPV to brachiocephalic vein via vertical vein
8	C + C	Type I	RIPV and RSPV to a confluence draining to IVC	LIPV and LSPV to a confluence to brachiocephalic vein via VV
9	CS + VV	Type II	RIPV, RSPV and LIPV to SC	LSPV to brachiocephalic vein via VV
10	CS + VV	Type II	RIPV, RSPV and LIPV to SC	LSPV to brachiocephalic vein via VV
11	SVC + RA	Type II	RSPV to SVC	LIPV, LSPV and RIPV to RA
12	CS + C	Type I	RIPV and RSPV to CS	LSPV and LIPV to a confluence to brachiocephalic vein via VV
13	CS + VV	Type II	RIPV, RSPV and LIPV to SC	LSPV to brachiocephalic vein via VV

*CS* coronary sinus, *LA* left atrium, *LAA* left atrial appendage, *LIPV* left inferior pulmonary vein, *LSPV* left superior pulmonary vein, *RA* right atrium, *RIPV* right inferior pulmonary vein, *RSPV* right superior pulmonary vein, *SVC* superior vena cava, *VV* vertical vein

Surgical correction in cases of type I TAPVC (the fourth and twelfth patients) involved complete unroofing of the CS wall into the LA and patch closure of the atrial septal defect (ASD) (similar to treatment of the cardiac type); then, the confluent was anastomosed to the LA. Surgical correction in the eighth patient involved anastomosing the left confluent section to the LA and the right confluent section to the back of the RA, followed by baffling to the LA to avoid the excessive tension involved in anastomosis to the LA. Surgical correction in cases of type II TAPVC (the first, third, fifth, sixth, seventh, ninth, tenth and thirteenth patients) involved complete unroofing of the CS wall into the LA, anastomosis of the VV to the LA or left atrial appendage (LAA) after transection, and closure of the VV near the innominate vein. In the second and eleventh patients, a Warden procedure was carried out, and the left pulmonary veins were rechanneled to the LA with baffling while closure of the ASD redirected the pulmonary veins to the LA (van Son procedure).

## Results

**Fig. 2:**
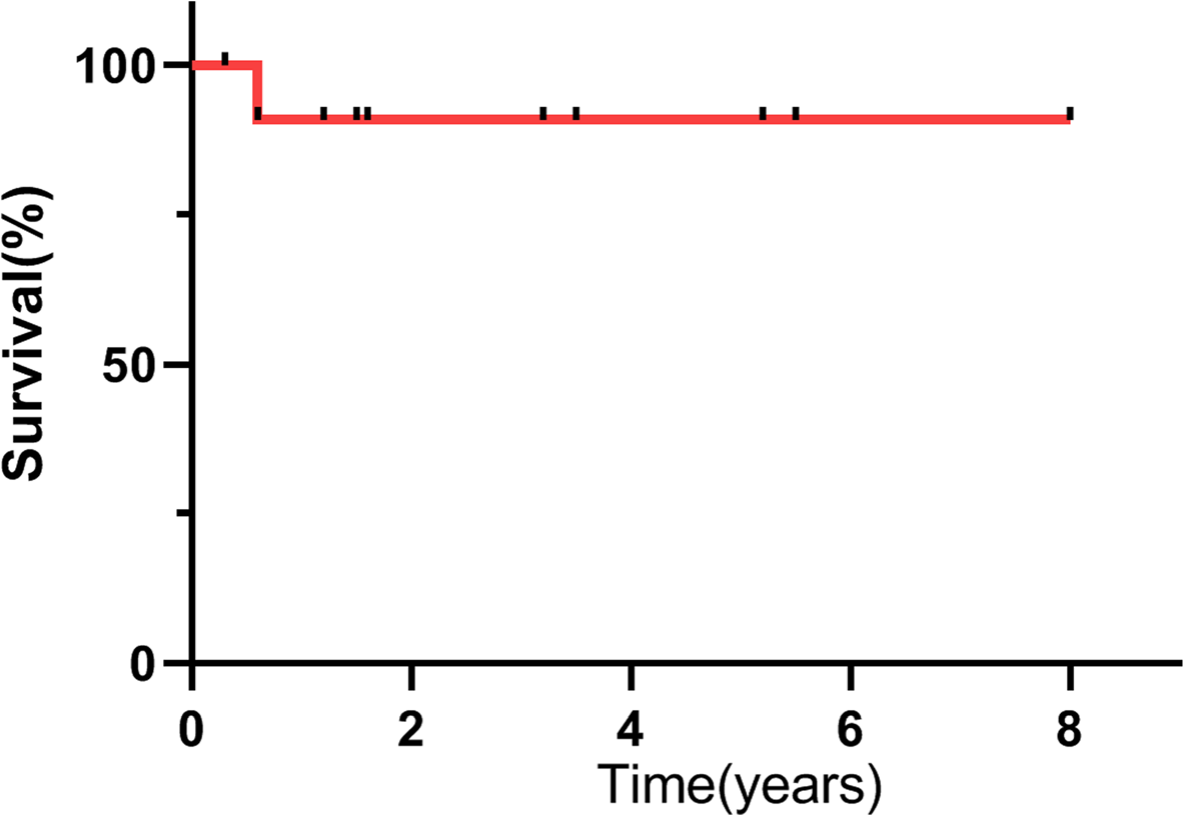
Kaplan-Meier survival curve for all patients

Hospital mortality was defined as mortality occurring less than 30 days after surgery or before discharge. All other deaths were defined as late mortality. ASD closure was performed in all patients. The median cardiopulmonary bypass time was 132.7 ± 25.1 min (range, 100 to 190 min). The cross-clamping time was 69.2 ± 14.4 min (range, 45 to 88 min). The second patient developed arrhythmia and pneumonia, the seventh, eighth and ninth patients developed low cardiac output syndrome, and the first and sixth patients developed pneumonia. Circulatory arrest was used in the third patient (7.7%, 1 of 13) during surgical repair. No anomalous pulmonary veins were left unrepaired at the time of surgery. The hospital mortality rate was 7.7% (1 of 13). The cause of early death was low cardiac output syndrome. There was 1 case (the third patient) of late mortality because of PVO 7 months after surgery. The patient developed metabolic acidosis and respiratory distress that required intubation. The parents refused surgical treatment when echocardiography showed pulmonary venous flow at the anastomotic of 2.9 m/s. No other reoperations for postoperative PVO were required (Table 3). Follow-up data were obtained for all survivors except the first patient at an average of 3.4 ± 2.2 years (5 months to 8 years) after surgery, and the remaining patients were asymptomatic at the last follow-up. The survival rates at 3 and 5 years were both 90.9% ± 8.7% (95% CI: 73.8–108%) (Fig. 2).

**Table 3:** Patient characteristics on operation

Patients	Cardiopulmonary bypass time (min)	Cross-clamping time (min)	Complications	Postoperative PVO	Follow-up
1	128	81	Pneumonia	NO	Lost
2	100	46	Arrhythmia, pneumonia	NO	Alive
3	149	45	Bronchomalacia, pneumonia	YES	Late death
4	151	77	None	NO	Alive
5	128	81	None	NO	Alive
6	149	68	Pneumonia	NO	Alive
7	190	85	Low cardiac output syndrome	NO	Alive
8	110	69	Low cardiac output syndrome	–	Early death
9	112	63	Low cardiac output syndrome	NO	Alive
10	113	58	None	NO	Alive
11	119	80	None	NO	Alive
12	157	88	Pneumonia	NO	Alive
13	119	58	None	NO	Alive

## Discussion

Mixed TAPVC is the least frequent subtype, accounting for 5 to 10% of patients with TAPVC in large series (our series is 6.4%). Because of the unpredictability of the pulmonary venous connections, mixed TAPVC is also the most problematic type and requires a combination of techniques, including the individualized anastomosis of pulmonary veins. An individually tailored operation should be formulated to successfully manage the highly variable anatomical pulmonary vein connections. Therefore, a correct preoperative understanding of the anatomy and an accurate description of all anomalously draining pulmonary veins are the first issues to be addressed [6].

Echocardiography can provide anatomical details regarding the position of anomalous pulmonary venous connections and the presence of venous obstruction in the majority of patients. In most cases, echocardiography is sufficient and preferable but sometimes can only identify two or three veins, which can lead to misdiagnosis. Some reports [7] have shown that the mixed type is the most difficult type to diagnose, with 4 (44.4%) patients who were diagnosed only after surgical examination [6]. In order to identify all four veins, computed tomography angiography should be applied, which can provide not only an accurate description of the pulmonary venous drainage pattern but also delineate the pulmonary vein anatomy in patients with suspected asymmetrical PVO, despite the radiation exposure [8, 9]. A merit of computed tomography angiography is three-dimensional reconstruction, which can provide a precise noninvasive visual description of the pulmonary vein connections [8, 9]. Cardiac catheterization is the third diagnostic method that can be considered. Due to the invasiveness, which will worsen the final outcome, it is not recommended as a routine examination [4]. We consider cardiac catheterization to be a choice in patients with stable hemodynamics and in whom both computed tomography angiography and echocardiography have failed, along with some other specific indications [4]. In our series, an accurate description of the drainage pattern for all four pulmonary veins was obtained in all 13 patients by echocardiography and computed tomography angiography.

Mixed TAPVC can be divided into 3 subtypes, as proposed by Chowdhury et al. Although accepted by most surgeons, this classification is little reference to the selection of the repair technique for the variants. Moreover, the surgical approach could be completely different even in cases of the same type.

Analysis of the published literature demonstrates a wide spectrum of technical aspects of the surgical repair [1–3]. How the pulmonary veins connect and the location of the drainage sites are determinant points of the surgical approach. We propose another classification based on the drainage sites to guide the surgical approach: (1) CS: Surgical correction in this group involved complete unroofing of the CS wall into the LA and patch closure of the ASD (modified Van Praagh technique) [6]. (2) Confluence: Surgical correction in this group involved sewing the site of confluence to the LA after transection. Anastomosis of the site of confluence to the LA requires accurate positioning to prevent stenosis [1, 3]. Sites of confluence on the right can be anastomosed to the back of the RA and baffled to the LA if the tension is too high when anastomosed to the LA. (3) VV: In this surgical approach, the VV is anastomosed to the LA or LAA directly after transection and closure of the VV near the innominate vein [1–4]. (4) RA: The surgery was performed by redirection of the pulmonary veins to the LA with baffling; while closure of the ASD redirected the pulmonary veins to the LA the ASD was extended according to the location of the pulmonary veins [3, 5, 6]. (5) SVC: In these patients, surgical correction of the pulmonary veins connecting to the superior vena cava was performed using the Warden procedure, in which the SVC was transected above the highest anomalous pulmonary vein, and the cardiac end of the SVC is oversewn. A baffle is then constructed in such a fashion that the right atrial orifice of the SVC is connected to the LA and closure of the ASD is achieved; then, the cephalad end of the SVC is sewn to the right atrial appendage. Many other surgical techniques have been described and proven to be useful and safe [6]. (6) ICV: In these patients, the veins were anastomosed to the back of the RA and then baffled to the LA; sometimes, the veins can be anastomosed to the LA directly [10]. (7) BAVs: In this group, the anatomical variants were bizarre and required pulmonary venous rechanneling on an individualized basis. The operative details for each morphological subtype should apply to the surgical approach and even allow two or more approaches to be combined [3]. The classification we propose is based on the various combinations of drainage sites combined with the corresponding approaches. The “CS + VV” pattern was most frequent in our series (61.5%), and the same tendency was found in most other studies [3, 4].

Unlike the surgical results of mixed TAPVC patients described in other studies, the statistical analyses in our series showed a good outcome regarding early mortality, with an occurrence rate of 7.7%. The pulmonary venous flow at the site of anastomosis was less than 1.2 m/s, as shown by postoperative echocardiography, which is considered adequate [11]. To achieve good outcomes, the following tips can be applied during anastomosis: (1) Sutureless repair, which has been reported to result in no mortality or reintervention, can be applied. Patients treated with sutureless repair revealed reasonable early and medium-term physiological tolerance, without the need for reintervention [12]. (2) Ensure precise geometric alignment and avoid tension, torsion, and rotation, making as large an anastomotic site as possible. (3) Maintain patency of the foramen ovale in patients with moderate-to-severe pulmonary hypertension for decompression of the right-sided chambers in the event of a pulmonary hypertensive crisis.

There are many limitations to our research. This was a retrospective single-center study, and the sample size was small. Additionally, the types of mixed TAPVC in our series are the more common types, unlike those in some other studies, which presented difficulties during the operation,which also would have a worse outcome. The classification we proposed also has limitations, it cannot account for all potential sites of pulmonary vein drainage, and the more uncommon types are classified as BAVs in our classification.

## Conclusion

In summary, mixed TAPVC is a comparatively rare variant of TAPVC. The diagnosis and accurate description of the pulmonary venous anatomy has important implications in the surgical treatment, and the variable geometry of multiple sites of pulmonary venous confluence is challenging. Echocardiography combined with computed tomography angiography is sufficient in most cases. The surgical scheme is determined by the drainage sites of the pulmonary veins. Therefore, we propose another classification on the basis of the drainage sites to guide the surgical approach. Operative survivors can achieve good long-term survival similar to that of patients with other forms of TAPVC if a suitable approach is applied to allow the anastomosis to be unobstructed.

## Data Availability

The data and materials in the manuscript are available, and the original data for the relevant results is owned by myself and can be contacted if it’s needed.

## Abbreviations

TAPVC: Total anomalous pulmonary venous connection
PVO: Pulmonary venous obstruction
CS: Coronary sinus
C: Confluence
VV: Vertical vein
IVC: Inferior vena cava
SVC: The superior vena cava
RA: Right atrium
BAVs: Bizarre anatomical variants
LAA: Left atrial appendage
LA: Left atrium
LIPV: Left inferior pulmonary vein
LSPV: Left superior pulmonary vein
RIPV: Right inferior pulmonary vein
RSPV: Right superior pulmonary vein
